# Thermophysical Properties of Undercooled Alloys: An Overview of the Molecular Simulation Approaches

**DOI:** 10.3390/ijms12010278

**Published:** 2011-01-10

**Authors:** Yong J. Lv, Min Chen

**Affiliations:** 1 Key Laboratory of Cluster Science, Ministry of Education of China, Department of Physics, Beijing Institute of Technology, Beijing 100081, China; E-Mail: yongjunlv@tsinghua.edu.cn; 2 Department of Engineering Mechanics, Tsinghua University, Beijing, 100084, China

**Keywords:** thermophysical property, undercooling, liquid metal and alloy, molecular dynamics simulations, Monte Carlo simulations

## Abstract

We review the studies on the thermophysical properties of undercooled metals and alloys by molecular simulations in recent years. The simulation methods of melting temperature, enthalpy, specific heat, surface tension, diffusion coefficient and viscosity are introduced and the simulated results are summarized. By comparing the experimental results and various theoretical models, the temperature and the composition dependences of the thermophysical properties in undercooled regime are discussed.

## 1. Introduction

The thermophysical properties of undercooled alloys play an important role in understanding the thermodynamic and dynamic behaviors of metastable liquids. They attract growing interest in both application and fundamental fields. In the application, the knowledge of thermophysical properties is crucial for controlling the crystal growth process and preparing the well-performance alloys. The formation capability of amorphous alloys, for example, is determined by the thermodynamic and dynamic behaviors of related melts, and the thermophysical parameters, including enthalpy of mixing and entropy of mixing, are necessary for designing amorphous alloys [1–6]. From the point of view of fundamental research, the validity of solidification theories, in particular for the rapid solidification process under highly undercooled conditions, largely depends on the accuracy of thermophysical data [7–10]. More importantly, the temperature and pressure dependences of the thermophysical properties give us an indication to understand the nature of novel phase transitions hidden in the undercooled environment and are helpful in exploring unknown physical and chemical processes.

The thermophysical properties refer to the multidisciplinary researches including physics, chemistry, materials science, nanoscience and nanotechnology. In the last three years, there have been nearly 100 research papers published in various journals per year [11], in which the mostly related thermophysical properties focus on the density, surface tension, specific heat, heat capacity, thermal and mass diffusion coefficient, viscosity and thermal conductivity. The involved systems involved range from pure metals, binary alloys to complicated ternary alloys, and the alloy types include the transition metal alloys, semiconductor-metal alloys, superalloys and others.

Experimental measurements and computer simulations are two major approaches to obtain the thermophysical properties of undercooled alloys. The space experiments can provide excellent containerless and ultrahigh vacuum environments that are necessary for achieving high undercooling [12–16]. Therefore, measurements based on space experiments are preferable. However, the complicated manipulation of space experiments as well as the high cost seriously limits its application. Up to now, there is rather limited thermophysical data from space experiments and data on the related alloy systems is also restricted. In contrast, a large number of experimental measurements are performed under the ground-based conditions. In order to avoid the contamination from container walls and enhance the undercoolibility, various levitation technologies including the electromagnetic [17–25], aerodynamic [26–31], electrostatic [32–34] and acoustic levitation [35–38] are employed. These experimental technologies are beneficial in realizing the high undercooling of metals, and simultaneously measure the thermophysical properties with high accuracy.

Although experimental measurement of thermophysical properties is intensively performed, constraint is still shown. Firstly, the measurable thermophysical properties from experiments are limited, and the measurements of some dynamic quantities like diffusion coefficient in experiments are not easy. Secondly, for some special alloys, achieving high undercooling is very difficult in experiments. These limitations prompt researchers to utilize computer simulations in studying thermophysical properties.

Computer simulations can easily maintain various metastable states such as high undercooling and high pressure by simply choosing appropriate simulation parameters. Moreover, it is allowable to calculate the thermophysical properties that are difficult to measure in experiments and to investigate the thermophysical phenomena on an atomic scale. With the rapid development of computation capacity in recent years, the large-scale and long-time simulations are widely adopted to reproduce more accurate thermodynamic and dynamic processes. Computer simulation has become one of the most important tools in studying thermophysical properties and behaviors. In this field, the major simulation methods include the classical molecular dynamics (MD) simulations, the Monte Carlo (MC) simulations and the *ab initio* molecular dynamics calculations. The MD and MC simulations are specialized disciplines of computer simulations of atomic and molecular scale system evolvement based on statistical mechanics. Up to now, MD and MC simulations are widely employed to calculate various thermophysical properties including density [39–45], specific heat [39–44,46–53], surface tension [44,48,54–57], diffusion coefficient [58–63] and viscosity [59,64–66], and referred systems cover most common metals or alloys. The validity of MD and MC simulations largely depends on the potential function. Most of the potentials are characterized by empirical or semi-empirical descriptions of interactions, which are usually obtained by fitting with corresponding experimental results. In some cases, these potential functions are not sufficient to accurately represent the thermodynamic or dynamic features. The *ab initio* MD method is an alternative, which is derived from the first principles based on the density functional theory and is independent of empirical parameters. Because of the large computational cost, this method is traditionally applied to the prediction of structures of smallscale systems. However, great improvements in computation capacity in recent years also permits the application of *ab initio* MD in the studies of thermophysical properties, and even some dynamic properties like viscosity that require long time relaxation are allowed [67–70].

In this paper, we focus on the thermophysical properties of undercooled alloys using MD simulations. We review the major methods and results in recent years and carry out comparisons with the experiments. In the second section, we introduce the methods of molecular simulations in studying thermophysical properties of liquid alloys. In the third part, we give a detailed summary of simulated results and comparisons with experimental results. Lastly, a conclusion is provided.

## 2. Molecular Simulations Approaches

Due to the size effect and artificial quenching process, the molecular dynamics (MD) and Monte Carlo (MC) simulations can easily produce a highly undercooled liquid, and thus are widely applied to study the thermophysical properties and the metastable phase transition of undercooled liquids at atomic level.

The validation of MD and MC simulations for predicting the thermophysical properties of undercooled melts largely depends on the potential function. For metal or alloy systems, the embedded atomic method (EAM) and the modified embedded atomic method (MEAM) are most preferred. EAM potential is firstly proposed by Daw and Baskes [71,72]. This method is based on the density functional theory and treats each atom as an impurity in the host of other atoms. The total energy consists of two parts: The embedding energy and the short-range electrostatic pair potential,

(1) $$E_{tot}=\sum_{i}F_{i}(\rho_{h,i})+\frac{1}{2}\sum_{j\neq i}\phi_{ij}(r_{ij})$$

where *r_ij_* and *ϕ_ij_* are the distance and the short-range pair potential between atoms *i* and *j*. (*ρ_h_*_,_*_i_*) *F_i_* *ρ* is the embedding energy of atom *i*, and *ρ_h_*_,_*_i_* denotes the electron density of the host without atom *i*, which is approximated to a sum of the atomic electron density of *j* atom at a distance *r_ij_* from site *i*,

(2) $$\rho_{h,i}=\sum_{j\neq i}\rho_{ij}^{a}(r_{ij})$$

The parameters in EAM potential are determined by fitting the lattice constants, elastic energy, sublimation energy and vacancy-formation energy of perfect crystals that are obtained from experiments or first-principle calculations. The EAM potential shows a good agreement with experiments in studying the bulk, surface properties and defects in crystals, and thus is widely adopted in the MD simulation of metal or alloy systems. The EAM was first applied in the studies of liquid metals by Foiles [73] who achieved good agreement between the EAM simulations and experiments in the structure factor of some transition metals. After that, the EAM has been used extensively to study the structural, thermodynamic, and dynamic properties of liquid metals and alloys. In the original EAM the embedded energy and pair potential are given in unanalytical forms, which causes some inconvenience in calculations and are unfavorable to the extension of this method. As a modification, the analytical EAM is developed based on the EAM. In the initial version, the total energy of a system is approximated to follow the equation of state of Rose *et al.* and the pair potential is assumed to be an analytical form [74]. As a result, the embedded energy has a form of a tabulate function of the host electron density. Following the work, several analytical EAM models are proposed to improve the EAM [75–77]. The EAM has been successfully applied to the face-centered cubic (fcc) and transition metals and is also used in some simulations of body-centered cubic (bcc) metals.

In the cases of silicon and germanium, Baskes *et al.* modified the EAM by taking the bonding directionality into consideration [78,79]. The host electron density in the modified version includes the angular dependent contributions, and this model is called modified EAM (MEAM). Baskes *et al.* attempted to reproduce bulk properties and surface phenomena of various metals and semiconductors with fcc, bcc, hexagonal close packed (hcp), as well as diamond structures, using the uniform potential. In fact, the simulated results show that the MEAM provides good descriptions of bulk and surface properties of bcc metals, semiconductors and their alloys.

In the MEAM, the embedded atomic potential is written as the following form,

(3) $$F_{i}(\rho_{i})=A_{i}E_{i}^{0}\frac{\rho_{i}}{Z_{i}}\text{ln}\left(\frac{\rho_{i}}{Z_{i}}\right)$$

where *ρ_i_* is the background electron density, *E_i_*^0^ is the cohesive energy of the reference structure, *Z_i_* is the number of nearest neighbors of reference structure, and *A_i_* is an adjustable parameter. The form of the background electron density varies with the element type, for example,

(4) $$\rho_{i}=\frac{\rho_{i}^{\left(0\right)}}{\rho_{i}^{0}}\frac{2}{1+\text{exp}(-\Gamma_{i})}\;\text{for Si},\text{Ge},\text{Sn}$$

(5) $$\rho_{i}=\frac{\rho_{i}^{\left(0\right)}}{\rho_{i}^{0}}\sqrt{1+\Gamma_{i}}\;\text{for Ni},\text{Au},\ldots$$

here *ρ_i_*^(^*^k^*^)^ is the partial electron density and

(6) $$\Gamma_{i}=\sum_{k=1}^{3}t_{i}^{\left(k\right)}{\left(\frac{\rho_{i}^{\left(k\right)}}{\rho_{i}^{\left(0\right)}}\right)}^{2}$$

In the EAM, the electron density is given by a linear superposition of the spherically average atomic electron density. The major modification in the MEAM is that the electron density involves the angular contributions besides the spherically symmetric partial electron density, *k* = 0–3 corresponding to *s*, *p*, *d*, and *f* symmetry, respectively,

(7a) $$\rho_{i}^{\left(0\right)}=\sum_{j\neq i}\rho_{j}^{a(0)}$$

(7b) $${\left(\rho_{i}^{\left(1\right)}\right)}^{2}=\sum_{\alpha =1}^{3}{\left[\sum_{j\neq i}x_{ij}^{\alpha }\rho_{j}^{a(1)}\right]}^{2}$$

(7c) $${\left(\rho_{i}^{\left(2\right)}\right)}^{2}=\sum_{\alpha ,\beta =1}^{3}{\left[\sum_{j\neq i}x_{ij}^{\alpha }x_{ij}^{\beta }\rho_{j}^{a(2)}\right]}^{2}-\frac{1}{3}{\left[\sum_{j\neq i}\rho_{i}^{a(2)}\right]}^{2}$$

(7d) $${\left(\rho_{i}^{\left(3\right)}\right)}^{2}=\sum_{\alpha ,\beta ,\gamma =1}^{3}{\left[\sum_{j\neq i}x_{ij}^{\alpha }x_{ij}^{\beta }x_{ij}^{\gamma }\rho_{j}^{a(3)}\right]}^{2}$$

where *x_ij_^α^* *r_ij_^α^* *r_ij_* = (*α* = *x*, *y*, *z*), *r_ij_* is the distance between atom *i* and *j. ρ_i_^a^*^(^*^k^*^)^ represents electron density contributed from atom *j* and shows an exponential decay with the distance. Similar to the EAM, the most parameters in the MEAM are determined by fitting the elastic properties of crystals from experiments or first-principle simulations. In the original MEAM, the interactions confined in the nearest-neighbor atoms are considered. The following modified versions are extended to second nearest-neighbor atom interactions (2NN MEAM) for overcoming the discrepancy in reproducing the surface energy of low-index surface that is deemed to be a result of neglecting the second nearest-neighbor atoms interactions [80,81].

The MEAM has been intensively used to describe some surface phenomena and behaviors including structure, thermodynamic and dynamic properties. It is also applied to some liquid metals. Ravelo and Baskes [82] simulated thermodynamic properties of *α*, *β*, and liquid phases of tin using a single MEAM potential, and have good agreement with experiments. Cheren [83] realized the nucleation of pure Ni during the quenching process using the MEAM potential, which is absent using the EAM potential, suggesting the validation of MEAM potential in describing the liquid characteristics. Recently, the application of the MEAM is extended to liquid alloys. Considering the fact that the parameters used in the MEAM are derived from the fits of crystal data, how to determine effectively the parameters suitable to liquid systems, in particular liquid alloys, is still an open issue.

### 2.1. Melting Temperature

The melting temperature is thermodynamically defined as the temperature at which the Gibbs free energies of liquid and solid are identical,

(8) $$G^{S}(P,T_{m})=G^{L}(P,T_{m})$$

Due to the influence of the interfacial energy, the values calculated by directly heating the simulation system with the periodical boundary conditions are usually larger than the experimental results. Therefore, the liquid/solid interface should first be constructed properly in simulations.

**Figure 1:**
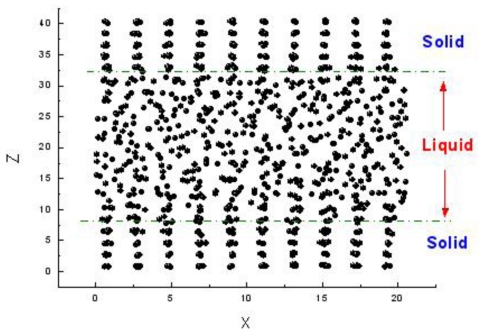
Model of sandwich structure for calculating melting point.

**Figure 2:**
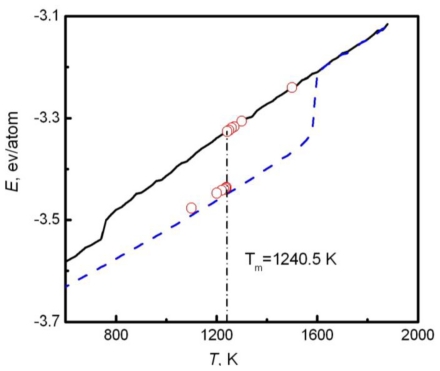
Internal energy of AuCu_3_ alloy *versus* temperature [41].

There are three methods to simulate the melting point. The first one is the sandwich method [84–86]. Two solid and one liquid subsystems are first equilibrated at different temperatures. Then the two solid systems sandwich the liquid layer and construct a solid-liquid-solid interlayer structure along the *Z* direction, as shown in Figure 1. The periodical boundary conditions are applied in the *X* and *Y* coordinate directions. In simulations, the whole system is annealed at a certain temperature, and the structural evolution of the interlayer is monitored during this process. If the sandwich structure completely melts into the liquid state, the current temperature is higher than the melting point, and contrariwise lower than the melting point. If the liquid/solid structure can stably exist, the temperature is approximated to the melting point. For example, Figure 2 shows the internal energy of a sandwich AuCu_3_ system *versus* temperature [41]. The solid and dashed lines represent cooling and heating processes from initial liquid and solid systems respectively, and the circles are the results from the sandwich method. Due to the extremely high cooling and heating rates, the crystallization and melting temperatures obtained from initial pure liquid or solid are far from the equilibrium melting point. Instead, the sandwich method can bypass heating and cooling processes in single-phase MD simulations and accurately produce the melting point. The difficulty of this method is that several trivial simulations are required in order to confirm the coexistence of liquid and solid phases, in particular in the region near the melting temperature.

The second method is the moving interface method [41]. At first, a liquid/solid interface is constructed at a desired temperature. After beginning the simulations, the moving velocity of interface is measured at the current temperature and then the temperature is changed with the interval *ΔT*.

The solid and dashed lines describe the cooling and heating from initial liquid and solid systems. The circle symbols predict the melting point with the sandwich method to repeat the measurement. Given enough measurements, we can plot a velocity-temperature curve, and extrapolate the moving velocity to zero, so the corresponding temperature is approximately regarded as the melting point. Similar to the sandwich method, this method requires many trials at different temperatures. The third one is the NVE ensemble method [87,88]. Similar to the second method, the liquid/solid double-layer structure is first set up near the melting point. Then, the structural relaxation of the system is performed in a constant volume, particle number, and energy (NVE) ensemble. If the system is initially assigned to a higher temperature than the melting point, the solid phase will partially melt. This process consumes the latent heat, and converts some of the kinetic energy into potential energy, resulting in the decrease in temperature. When the system is finally stabilized, the corresponding temperature is approximated to the melting point. Similarly, if the initial temperature is lower than the melting point, it will evolve toward the melting temperature from below. In contrast to the former two methods, the NVE ensemble method can determine the melting temperature in a single simulation. However, it is noted that the initial temperature should be set as near the melting temperature as possible. In fact, the NVE ensemble method integrated with the moving interface method is more advisable for calculating melting temperature. A rough estimate is performed using the moving interface method, and then a fine calculation is undertaken by the NVE ensemble method.

### 2.2. Specific Heat

Specific heat is a fundamental parameter in thermophysics, and plays an important role in understanding the thermodynamic and dynamic behaviors of liquids. It is also widely studied by MD simulations, particularly for undercooled liquids. The simulated systems involve the pure metals, binary and even ternary alloys.

In general, there are two methods used in calculating the specific heat. The first one is the statistical average of the energy fluctuation [89]. The expression of specific heat depends on the ensemble adopted in the simulations. In the canonical ensemble (constant particle number, volume and temperature), the isovolumetric specific heat is defined as

(9) $$C_{V}=\frac{N_{a}}{k_{B}T}\langle \delta E^{2}\rangle$$

where 〈*δE*^2^〉 = 〈*E*^2^〉 *−* 〈*E*^2^〉, *N_a_* is the particle number of systems. In the micocanonical ensemble (constant particle number, volume and energy), 〈*δE*^2^〉 = 0, and the relevant fluctuations to consider are those of kinetic energy *E_k_* or potential energy *E_u_*. Thus the isovolumetric specific heat is

(10) $$C_{V}=\frac{3k_{B}}{2}{\left(1-\frac{2N_{a}\langle \delta {E_{k}}^{2}\rangle }{3{\left(k_{B}T\right)}^{2}}\right)}^{-1}$$

This method requires a large-size simulation system and relatively simple interactions, such as gas, so its application is relatively limited.

The second method is the derivative of the thermodynamic function. The isobaric specific heat is written as the differential of enthalpy,

(11) $$C_{p}={\left(\frac{\text{d}H(T)}{\text{d}T}\right)}_{p}$$

where enthalpy *H*(*T* ) = *E* + *PV* is readily calculated in MD simulations. This method is more popular in calculating the specific heat, and the estimated results are also in good agreement with experimental measurements.

### 2.3. Density

The density is calculated by the ratio of mass to volume in MD simulations. In the constant particle number, pressure and temperature (NPT) ensemble, the volume of system is sampled directly at a constant temperature. Its agreement with the experimental measurements depends on the potential function. This conclusion is driven for not only density but most thermophysical properties. Density is closely related to many thermophysical properties, and experimental measurements about it are intensively carried out. Therefore, density is considered to be a good candidate for the test of potentials.

### 2.4. Surface Tension

There are three methods to produce the surface tension of fluids in MD simulations. The first one is to construct a liquid-vapor surface in the simulations and directly calculate the surface tension from the mechanical expression. The surface tension can be expressed as an integral of the difference between the normal and tangential pressure tensor components over the surface

(12) $$\gamma =\int_{z_{l}}^{z_{v}}\text{d}z(P_{N}-P_{T})$$

where *z_l_* and *z_v_* are the arbitrary positions in the bulk liquid and vapor phases respectively, *P_N_* and *P_T_* are the normal and tangential components of pressure tensor. In general, the simulation model is a liquid film that has two symmetry surfaces. Therefore the above equation is converted into

(13) $$\gamma =\frac{1}{2}\int_{0}^{L_{z}}\text{d}z(P_{N}-P_{T})$$

If the film is divided into *N* slabs with the length of *L_z_/N* parallel to the *X*–*Y* panel, the integral can be transformed to the sum in these slabs. From the statistical mechanical expressions, *P_N_* and *P_T_* in the *k*th slab are written as

(14a) $$P_{N}(k)=\langle n(k)\rangle k_{B}T-\frac{1}{V}\langle {\sum_{i,j}}^{\left(k\right)}\frac{z_{ij}^{2}}{r_{ij}}\varphi^{\prime }(r_{ij})\rangle$$

(14b) $$P_{T}(k)=\langle n(k)\rangle k_{B}T-\frac{1}{V}\langle {\sum_{i,j}}^{\left(k\right)}\frac{1}{2}\frac{\left(x_{ij}^{2}+y_{ij}^{2}\right)}{r_{ij}}\varphi^{\prime }(r_{ij})\rangle$$

where *n*(*k* ) is the number density of the *k*th slab, *V* = *L_x_* *· L_y_* *· L_z_* */ N* is the volume of a slab, *r_ij_* is the distance between particles *i* and *j*, *φ* (*r_ij_*) is the interaction potential. Therefore, Equation 13 has the following form [90],

(15a) $$\gamma =\frac{1}{2}\sum_{k=1}^{N}\gamma (k)$$

(15b) $$\gamma (k)=\frac{1}{L_{x}L_{y}}\langle {\sum_{i,j}}^{\left(k\right)}\frac{\frac{1}{2}(x_{ij}^{2}+y_{ij}^{2})-z_{ij}^{2}}{r_{ij}}\phi^{\prime }(r_{ij})\rangle$$

**Figure 3:**
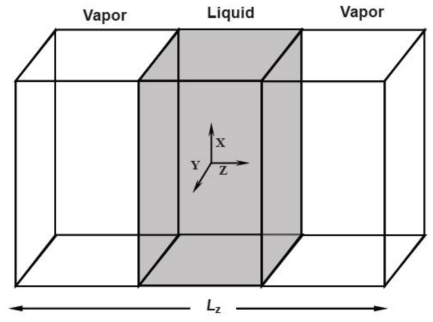
A rectangular simulated cell with a liquid film in the middle and vapor on each side of the cell.

In the MD simulations, the liquid-vapor surface is constructed as follows: A NPT system with the periodical boundary conditions along the three coordination directions is set up first and equilibrated, then the periodical boundary condition in the *Z* direction is withdrawn and the simulation box is prolonged in the *Z* direction, forming two vacuum regions sandwiching the liquid phase. After equilibrating in the NVT ensemble, two surfaces form. Figure 3 shows a schematic diagram of the simulation region.

The second method is derived from the thermodynamic expression of surface tension, namely, the additional free energy required by forming a new surface. One of these operations was carried out by Miyazaki *et al.* [91], in which the free energy for reversibly creating a surface in the bulk liquid was calculated using the MC simulation. Generally speaking, the first approach suffers from rather high fluctuation and statistical uncertainty, and the second method incurs additional complexity in the performance.

A bulk system is assumed to be split into two parts, and the difference of the free energy during this process is equal to the surface free energy, which is also known as cohesive energy. Therefore, the third method to simulate the surface tension, calculates the cohesion work of the liquid. This method was adopted by Padday and Uffindell [92] to calculate the surface tension of n-alkanes, as well as the interfacial tension between the n-alkanes and water. Lu [93] applied the method to calculate the surface tension of liquid argon, nitrogen and oxygen by summing the interaction energy across an assumed surface in the MC simulation. Chen *et al.* [54–56] extended the method to the liquid metal and alloy systems. They calculated the surface tension of liquid nickel, cobalt and Ni-Cu alloy using the MC simulations and the results showed reasonable agreement with experimental values.

**Figure 4:**
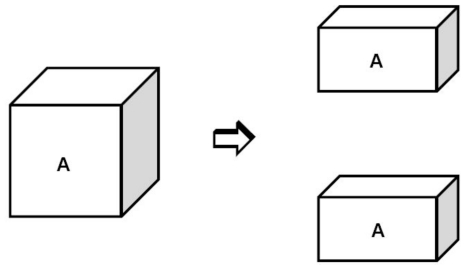
Calculation of surface tension by separating the liquid.

The simulated model is shown in detail in Figure 4. The bulk liquid phase *A* is separated into two liquid subsystems with equal volumes. During this process, two surfaces are created and the change of total free energy is equal to the sum of the surface energy of the two surfaces. The work of the cohesion *W_AA_* is defined as the work required to pull the liquid bulk system *A* apart, thus

(16) $$\Delta G=2\gamma_{A}=W_{AA}$$

Note that the equilibration of the surface region is not taken into account during the process of separation. It is assumed that the formation of surface phase cannot influence the thermodynamic behavior of the internal bulk phase. The quantity of heat exchange for obtaining equilibrium is *Q*, the entropy increase during the surface formation is *S*, and Equation 16 is modified as

(17) $$\Delta G=2\gamma_{A}=W_{AA}+Q-TS$$

In the calculation of the surface tension of n-alkanes, it is found to be reasonable to neglect the last two terms in Equation 17 [92]. Chen [54,55] extended the conclusion to the metal systems, and the surface tension is approximated by statistically calculating the free energy difference.

### 2.5. Diffusion

Diffusion is one of the most important research topics of liquid physics. It closely relates to many physical and chemical phenomena associated with mass transfer in liquid systems. During the solidification of alloys, the solute diffusion in front of the liquid/solid interface plays a key role in quantitative prediction of microstructural morphologies. For example, the stability of the solid/liquid interface in the directional solidification depends on the diffusion coefficient and growth velocity, and the former directly determines the microstructural evolution of the interface from planar to cells and final dendrites.

In general, the diffusion refers to all types of mass transporting processes, and thus shows a diversity of definitions. The simplest diffusion mode is self-diffusion. The mass transfer of the pure liquid metals can be described by this diffusion mode. In the more general definition, the self-diffusion is regarded as a kind of random-walk motion of a tagged particle of species *i* in a multi-component system and its velocity autocorrelation function is expressed by,

(18) $$C_{i,self}=\frac{1}{N_{i}}\sum_{j=1}^{N_{i}}\langle {\vec{u}}_{j}^{\left(i\right)}(t)\cdot {\vec{u}}_{j}^{\left(i\right)}(0)\rangle$$

where *u⃗_j_*^(^*^i^*^)^ (*t*) and *u⃗*_(_*_i_*_)_*^j^* (0) denote the velocity vector of particle *j* of species *i* at time *t* and 0 respectively, *N_i_* is the number of *i* particles, and 〈*·*〉 is the ensemble average. Correspondingly, in the equilibrium thermodynamics, the self-diffusion coefficient is calculated by means of the Green-Kubo relation that integrates over the autocorrelation function [94],

(19) $$D_{i,self}=\frac{1}{3}\int_{0}^{\infty }\langle {\vec{u}}_{j}^{\left(i\right)}(t)\cdot {\vec{u}}_{j}^{\left(i\right)}(0)\rangle \text{d}t$$

Alternatively, the self-diffusion coefficient can also be defined by the long time limit of the mean-squared displacement (MSD), namely the Einstein’s equation [89],

(20) $$D_{i,self}=\frac{1}{6N_{i}}\underset{t\to \infty }{\text{lim}}\sum_{j=1}^{N_{i}}\frac{1}{t}\langle |{\vec{r}}_{j}^{\left(i\right)}(t)-{\vec{r}}_{j}^{\left(i\right)}(0){|}^{2}\rangle$$

where *r⃗_j_*^(^*^i^*^)^(*t*) and (0) *r⃗_j_*^(^*^i^*^)^ are the positions of particle *j* of species *i* at time *t* and 0 respectively. The two self-diffusion coefficients above are equivalent and are fundamental of computer simulations of self-diffusion coefficient.

Like the case of the mass transport of absorbed molecules in porous materials, another diffusion process, based on the mass flux driven by concentration gradients, is defined, in which the diffusion flux is expressed by the well-known Fick’s law,

(21) $$J=-D_{t}(c)\nabla c$$

here *D_t_* is defined as Fickian diffusion coefficient or transport diffusion coefficient. Compared with the diffusion in a single-component system, the Fickian diffusion in a mixture shows more complicated. The interplay between different species becomes pronounced, and even some abnormal diffusion phenomena are observed. For instance, the experiments have demonstrated that N_2_ molecules diffuse against the concentration gradient in an ideal gas mixture [95,96]. Several theoretical models are proposed to overcome the difficulty. Based on the irreversible thermodynamics, the diffusion driven by the chemical potential gradients is developed. In this model, the diffusion flux, *J* is given by a formula related to chemical potential gradients [96,97],

(22) $$J_{i}=-\sum_{j=1}^{n}L_{ij}(\mu_{1},\cdots \mu_{n})\nabla \mu_{j}$$

where *L_ij_* is the symmetric matrix of Onsager transport coefficients, *n* is the number of species, *∇μ_j_* is the chemical potential gradient of species *j*. The flux can also be independently given by the Fick’s law in multi-component systems as a function of concentration gradients,

(23) $$J_{i}=-\sum_{j=1}^{n}D_{ij}(c_{1},\cdots c_{n})\nabla c_{j}$$

where the diffusion coefficient *D_ij_* is not symmetric and is called the Fickian diffusion coefficient or the binary transport diffusion coefficient. Maxwell and Stefan [97–99] proposed another model based on the kinetics process of ideal gas mixtures for describing the diffusion process above. They assumed that the chemical potential gradient is balanced by the frictional drag and the deviation of the force balance will lead to the diffusion flux. The frictional drag is proportional to the relative velocity of species *i* and *j*, *u_i_* − *u_j_* and mole fraction *c_j_*, then we get

(24) $$-\frac{1}{RT}\nabla \mu_{i}=\sum_{j=1;j\neq i}^{n}\frac{c_{j}(u_{i}-u_{j})}{D_{ij}}$$

where *Ð_ij_* is the Maxwell-Stefan (M-S) diffusion coefficient and can be regarded as an inverse drag coefficient. The matrix *Ð_ij_* is symmetric on the basis of the Onsager reciprocal relations. For the ideal mixture, *Ð_ij_* is independent of the composition, otherwise it is not. It is noted that the diffusion processes in the multi-component systems described in Equations 22–24 are mathematically equivalent, and there are no approximate relations among them.

For a binary system, the diffusion seems to be much simplified. The Fickian diffusion coefficient is related to the M-S diffusion coefficient by only multiplying a factor,

(25) $${Ð}_{12}=\frac{D_{12}}{\Omega }$$

where Ω is the thermodynamic correction factor that is related to the activity *a*_1_ of species 1,

(26) $$\Omega ={\left(\frac{\partial \text{ln}a_{1}}{\partial \text{ln}c_{1}}\right)}_{T}$$

In MD simulations, the self-diffusion coefficient can be obtained using Equations 19 or 20. The M-S diffusion coefficient is calculated by the autocorrelation function of velocity flux [89],

(27) $${Ð}_{12}=\frac{1}{3Nc_{1}c_{2}}\int_{0}^{\infty }\langle \vec{J}(t)\cdot \vec{J}(0)\rangle \text{d}t$$

where the velocity flux, $\vec{J}(t)=c_{1}\sum_{i\in 2}{\vec{u}}_{i}(t)-c_{2}\sum_{i\in 1}{\vec{u}}_{j}(t)$, and *N* is the total number of particles.

M-S diffusion coefficient shows more difficult to measure in experiments due to its independence of concentration. Instead, an approximate relation is proposed for the binary system, namely, the Darken relation [100],

(28) $${Ð}_{12}=c_{1}D_{2,self}+c_{2}D_{1,self}$$

which relates the M-S diffusion coefficient to the self-diffusion coefficients, *D*_1,_*_self_* *D*_2,_*_self_*, and concentrations, *c*_1_, *c*_2_ of species 1 and 2. In fact, if the velocity cross correlations 〈*u⃗_i_* (*t*) · *u⃗_j_* (0) 〉, *i* ≠ *j* is negligible in Equation 27, the average M-S diffusion coefficient is accordingly simplified to the Darken relation as,

(29) $${Ð}_{12}=\frac{c_{2}}{3}\int_{0}^{\infty }\langle {\vec{u}}_{1}(t)\cdot {\vec{u}}_{1}(0)\rangle dt+\frac{c_{1}}{3}\int_{0}^{\infty }\langle {\vec{u}}_{2}(t)\cdot {\vec{u}}_{2}(0)\rangle dt$$

The Darken relation has been proved to operate well in some metals and organic systems, and provides an alternative way to obtain the M-S diffusion coefficients indirectly. Although this is so, the diffusion mechanism and diffusion data in the binary and multi-component systems still largely depend on the computer simulations.

### 2.6. Viscosity

#### 2.6.1. Equilibrium MD Simulations

Viscosity is associated with the pressure tensor within liquids. There are two approaches to calculate viscosity in the MD simulations, equilibrium MD (EMD) and non-equilibrium MD (NEMD) methods.

In the equilibrium MD method, the shear viscosity is calculated using the Green-Kubo relation, integrating the autocorrelation function of the pressure tensor [89]

(30) $$\eta =\frac{V}{k_{B}T}\int_{0}^{\infty }\langle \vec{P}(t)\cdot \vec{P}(0)\rangle \text{d}t$$

where *P*(0) and*P*(*t*) are the pressure tensors at time 0 and *t*. The pressure tensor is defined as

(31) $$P_{\alpha \beta }=\sum_{j=1}^{N}\left(\frac{p_{\alpha j}p_{\beta j}}{m_{j}}+r_{j\alpha }F_{j\beta }\right)\;\;\;(\alpha ,\beta =x,y,z)$$

where *P_αβ_* is an off-diagonal element (*α* ≠ *β*) of pressure tensor matrix. *p_αj_* and *p_βj_* denote the momenta of the *j*th particle along *α* and *β* directions, *r_jβ_* is the particle position component and *F_jα_* is the *α* component of force on the *j*th particle. The equilibrium MD method requires a relatively long simulation time to wait for the dynamic relaxation of the system. Especially under high undercooling conditions, the decay scope of the autocorrelation function rapidly increases and the calculation becomes more difficult. Instead, the non-equilibrium MD calculations are proposed.

#### 2.6.2. Non-Equilibrium MD Simulations

**Figure 5:**
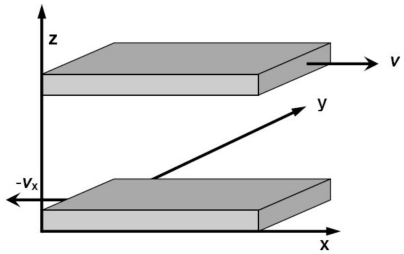
Schematic diagram of calculating viscosity of Couette flow.

Based on the definition of viscosity, the core idea of calculating viscosity in the non-equilibrium MD simulations is yielding a stable shear flow along a fixed direction. Figure 5 shows the simulation cell of Couette flow proposed by Lees and Edwards [101]. The periodical boundary conditions are applied in both *X* and *Y* coordinate directions, and two parallel walls are introduced perpendicular to the *Z* axis. Then, a constant slide velocity *v* *_x_* is exerted to the upper wall in the positive *x* direction, and an opposite and constant velocity −*v* *_x_* is applied to the lower wall. As a result, a thin and relatively stationary fluid layer forms close to each wall if the walls are rough enough, and finally a planar Couette flow is developed within the liquid confined between the two walls. In the fluid, the linear velocity profile is constructed and the shear rate is given by

(32) $$\sigma =\frac{{\text{d}v}_{x}}{\text{d}z}$$

The shear viscosity is equal to the ratio of pressure tensor *P_xy_* to shear rate *σ*,

(33) $$\eta =-\frac{P_{xy}}{\sigma }$$

If the two walls are fixed and the fluid is forced to flow between the two walls, a Poiseuille flow is obtained. From the exact solution of the Navier-Stokes and heat conduction equations, the velocity and temperature profiles of fluids are [102].

(34) $$v_{x}(z)=\frac{\rho gL_{z}^{2}}{2\eta }\left[\frac{1}{4}-{\left(z-\frac{1}{2}\right)}^{2}\right]$$

(35) $$T(z)=T_{w}+\frac{\rho^{2}g^{2}L_{z}^{4}}{12\lambda \eta }\left[\frac{1}{16}-{\left(z-\frac{1}{2}\right)}^{4}\right]$$

where *L_z_* is the channel width, *T_w_* is the wall temperature that is maintained constant in the simulations, *g_z_* is the external force driving the flow, and *λ* is the thermal conductivity. The periodical boundary conditions are held in the *x* and *y* directions. Dividing the simulation region into several slices parallel to the walls and calculating the mean flow velocity and temperature in each slice, the viscosity and thermal conductivity can be obtained by fitting the velocity and temperature profiles based on Equations 34 and 35.

In the problems mentioned above, a prominent character is the introduction of the physical walls. How to describe the wall realistically in simulations is the key to producing reasonable results. Another approach is that these transport phenomena are considered in a homogeneous system without the external wall, in which the equations of motion are modified to calculate the transport coefficients from the linear response theory. This approach is regarded more as a computation technique rather than the realistic description of the transport process [102].

**Figure 6:**
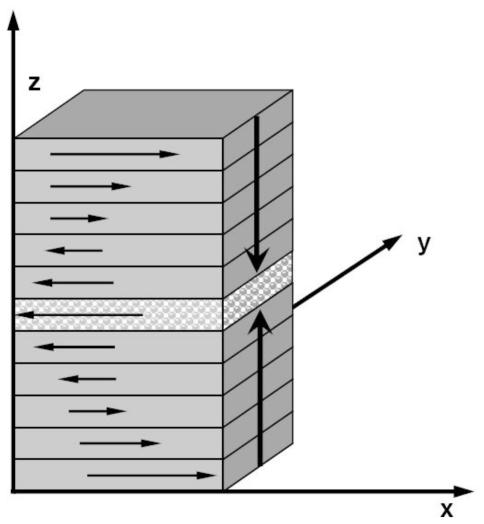
Schematic diagram of reverse non-equilibrium molecular dynamics (RNEMD) method.

Muller-Plathe [103–105] developed another non-equilibrium MD simulations method, the reverse non-equilibrium molecular dynamics (RNEMD) method to calculate various transport coefficients. Figure 6 plots a schematic diagram of the simulation cell. The simulation box is divided into *N* slices. After beginning the simulations, search the particles in the first slice (*M* = 1) and the center slice (*M* = *N*/2 + 1), and find out the one with the largest positive *x* component of the momentum in the first slice and the one with the largest negative *X* component of the momentum, then exchange the *X* component of velocity between the two particles. The procedure is repeated every fixed time step, and a stable shear momentum flow is finally developed. Assuming that the momentum change in each exchange is *Δp_x_*, the momentum flow, *j_z_* (*p_x_*) is given by

(36) $$j_{z}(p_{x})=\frac{\sum \Delta p_{x}}{2tA}$$

where *t* is the simulation time and *A* = *L_x_* *L_y_*. Müller-Plathe applied the method to liquid Ar, N_2_, water and hexane and yielded good agreement with the experimental results.

## 3. Simulated Results and Comparison with Experiments

### 3.1. Melting Temperature

Table 1 summarizes the simulated melting temperatures of Au, Cu, Au-Cu, Ni-Al and Al-Ni alloys at zero pressure. The accuracy of MD simulations varies with systems. For AuCu_3_ and Au-Cu alloys, agreement with the experimental values shows rather good, but the calculated melting point underestimates the experimental result by about 20% in Al_50_Ni_50_ alloy [106].

The accuracy of these methods depends largely on the potential function. The parameter, function forms and the truncation of potential are believed to be the important factors that influence the simulated results, but the system size shows independent. On the other hand, the experimental measurements of melting temperature are often influenced by the surface melting [107]. The melting process of metal initiates from the surface pre-melting, and the temperature corresponding to the surface melting is usually larger than the thermodynamic melting point, which is also a factor responsible for the discrepancy between experiments and simulations. With the development of computation technique, large scale simulations based on the first principle are also applied to the coexistence of liquid and solid metals, such as aluminum [108], and the results show a good agreement with other approaches.

### 3.2. Enthalpy and Specific Heat

#### 3.2.1. Pure Liquid Metal

**Figure 7:**
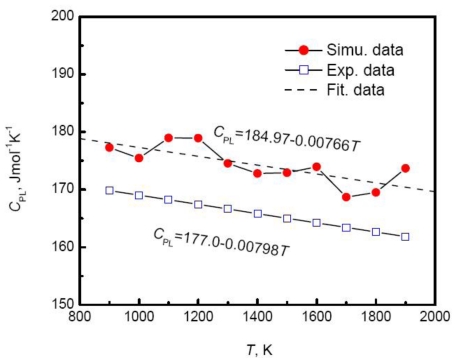
Specific heat of liquid copper *versus* temperature [109].

Massive simulated results show that the enthalpy of liquid metals linearly depends on the temperature. Han *et al.* [40] calculated the enthalpy of liquid cobalt using two sets of EAM parameters and found that the potential model importantly influences the accuracy of simulated results. Compared with the experimental measurements near the melting temperature, the proper simulations can control the error within 5%. Due to the linear temperature dependence of enthalpy, the corresponding specific heat is constant in the simulated temperature range. The result suggests that the specific heat in the limited undercooled region can be approximated by the value at the melting temperature. Further extensive experiments indicate that the specific heat actually increases slowly with the decrease of temperature in a wider temperature range. The temperature dependence of enthalpy may be more accurately described by a quadratic polynomial, and as a result the specific heat varies linearly with the temperature. Alternatively, the differential of the enthalpy is carried out at every discrete temperature instead of the fitted function, and then the variation of the specific heat with temperature is obtained by linearly fitting. Figure 7 displays the specific heat of liquid copper *versus* temperature using the above method. Although the specific heat obviously fluctuates, an overall linear relation is still ensured, and moreover shows a good agreement with the experiments. This fluctuation is believed to be a result of thermal fluctuation introduced by the MD method, and the increase of system size and optimization of potential parameters can weaken the fluctuation. Tables 2 and 3 summarize the simulated results of enthalpy and specific heat of some typical liquid metals.

#### 3.2.2. Binary Liquid Alloy

Similar to the pure liquid metals, the temperature dependence of enthalpy of most binary liquid alloys is also approximated by a linear function, and the composition does not show a remarkable influence on the temperature behavior. The enthalpies of Au_3_Cu, AuCu and AuCu_3_ as a function of temperature are given as follows [41],

(37a) $$H_{{Au}_{3}Cu}=-2.31358\times 10^{6}+(209.95\pm 0.66)\cdot T$$

(37b) $$H_{AuCu}=-2.86068\times 10^{6}+(262.79\pm 1.66)\cdot T$$

(37c) $$H_{{AuCu}_{3}}=-3.73859\times 10^{6}+(355.71\pm 1.90)\cdot T$$

**Figure 8:**
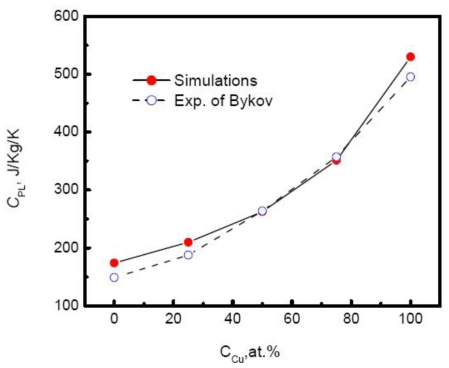
Specific heat of liquid Au-Cu alloys *versus* composition [41].

Although linear relationship between temperature and enthalpy is independent on the composition, the slope or specific heat increases with the increase of the Cu composition. Figure 8 plots the specific heat of Au-Cu alloy *versus* the composition. As the Cu composition increases, the specific heat of systems rises exponentially,

(38) $$C_{PL}=141.16+36.24\text{exp}(X_{Cu}/42.19)$$

**Figure 9:**
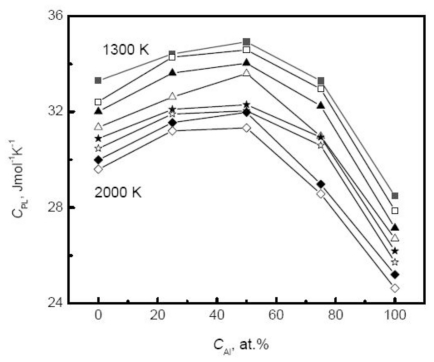
Specific heat of liquid Ti-Al alloys *versus* composition [39].

**Figure 10:**
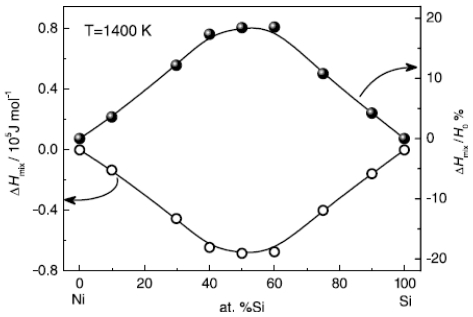
Simulated mixing enthalpy *H*_mix_ and *H*_mix_/*H*_0_ of liquid Ni-Si alloys. *H*_0_ is the enthalpy of the ideal mixture [42].

Moreover, the simulated results agree well with the experimental measurements above the melting temperatures by Bykov [113]. The composition dependence of specific heat shows more complicated and varies with the component of alloys. For many liquid alloys, the specific heat does not change monotonically with the composition. For example, the specific heat of Ti-Al alloys increases first to a maximum at the moderate composition 50% Al and then decreases with the increase of Al composition [39], as shown in Figure 9. Therefore, for binary systems, the Neumann-Kopp law, that intends to fit the thermophysical properties of the mixture using a linear combination of the values of various pure components, shows less effective. This deviation originates from the nature of liquid alloys. For most liquid alloys, the mixing effect exists, and the liquid systems cannot be regarded as ideal solutions. The mixing enthalpy is defined as the difference between the values of actual liquids and the assumed ideal mixture. Figure 10 shows the calculated mixing enthalpy of Ni-Si alloys at different compositions [42]. The enthalpy negatively deviates from the value of the ideal solution; in particular in the moderate composition range, the mixing enthalpy reaches the minimum.

Generally, the mixing enthalpy closely depends on the systems, which determines that it is difficult to propose a universal form to describe the composition dependent specific heat.

Besides the above alloys, Fe-Ni, Ni-Al and Ni-Mo alloys are also studied [46–48], and these data contribute to the development of theoretical researches in phase transition and liquid physics. Even the specific heat of some ternary alloys is also reported [52,114]. The determinant of EAM or MEAM parameters requires the relevant experimental or first-principle results, which shows more difficult for the complicated ternary systems. Therefore, the predicted values of ternary alloys need to be further examined.

### 3.3. Density

As the elementary thermophysical quantity, density of liquid alloys as well as its temperature and pressure dependences is crucial for describing thermodynamic state of liquids. For the undercooled melts, the difficulty of measuring the density lies in realizing the undercooling state. Therefore, the normal contact measurement of density shows impracticable. At present, the electromagnetic levitation technique, in combination with the image acquisition method, is widely employed in measuring density of undercooled alloys. The principle of measuring density follows the definition of density,

(39) $$\rho =\frac{M}{V}$$

In experiments, the conservation of mass is held and the mass of sample can be approximated by weighing the solidified sample after experiments. The key for the method is the accurate measurement of volume. In the ground-based experiments, the shape of droplets is deformed induced by the gravity and electromagnetic fields. The equilibrium shape is assumed to have rotational symmetry. Therefore, the droplet volume is expressed by,

(40) $$V=\frac{2\pi }{3}\int_{-1}^{1}r^{3}(\text{cos}\theta )\text{d}(\text{cos}\theta )$$

where *r* is the radial radius and *θ* is the polar angle. The radial radius can be written as a sum of Legendre polynomials, which is determined by fitting the captured images. It is experimentally found that the temperature dependence of the density of liquid metals follows a linear relation,

(41) $$\rho =\rho_{m}+\frac{\partial \rho }{\partial T}(T-T_{m})$$

where *ρ_m_* is the density of liquid metal or alloy at the melting temperature *T_m_*.

Compared with the experimental values above the melting temperature, the simulated densities of pure metals generally display a good agreement. Table 4 shows a comparison of the simulated and experimental (above melting temperature) values of some pure liquid metals.

The error between the simulations and experiments is less than 5% near the melting temperatures; particularly for liquid titanium, the error is controlled within 1%. In fact, for some special systems such as aluminum, germanium, the density seems to deviate from the linear relation in a wide temperature range. This deviation may be related to the existence of local ordering clusters in the undercooled region. Moreover, the alloys, such as Ni-Si [42] and Ti-Al [39], also show non-linear temperature dependences in some composition ranges. Instead, the quadratic polynomial as

(42) $$\rho =\rho_{m}+\frac{\partial \rho }{\partial T}(T-T_{m})+\frac{\partial^{2}\rho }{\partial T^{2}}{\left(T-T_{m}\right)}^{2}$$

is used to describe the temperature behavior.

**Figure 11:**
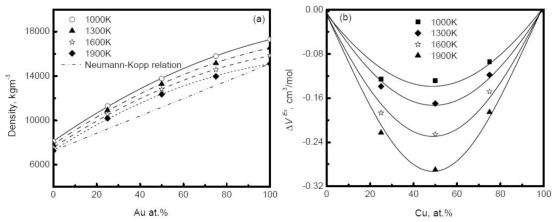
Density (**a**) and excess volume (**b**) of liquid Au-Cu alloy *versus* composition [41].

For an ideal binary mixture, the density can be represented by the Neumann-Kopp relation associated with the thermodynamic quantities of the two pure elements,

(43) $$\rho (T)=\frac{c_{A}M_{A}+c_{B}M_{B}}{c_{A}V_{A}(T)+c_{B}V_{B}(T)}$$

where *M_A_* and *M_B_* are the molar masses of *A* and *B* components, *V_A_* and *V_B_* are the molar volumes of *A* and *B* components respectively, and *c_A_* = 1− *c_B_* is the molar concentration. However, for most alloy melts, Equation 43 does not provide a reasonable description of the composition dependence of alloys. Figure 11(a) shows the simulated density of Au-Cu alloys as a function of composition at different temperatures [41]. The deviation of simulated density from the prediction of the Neumann-Kopp relation becomes more serious as the composition increases, and reaches a maximum in the moderate composition region. Similar behavior is also observed in experiments. For example, the density of Cu-Ni alloy positively deviates from the prediction of ideal solution model in the middle composition region [20]. The main reason for the deviation is the remarkable volume effect that occurs when two pure elements are melted into an alloy. A concept of excess volume *ΔV* is introduced to correct the density in the ideal solution model,

(44) $$\rho (T)=\frac{c_{A}M_{A}+c_{B}M_{B}}{c_{A}V_{A}(T)+c_{B}V_{B}(T)+\Delta V}$$

where

(45) $$\begin{array}{l} \Delta V=V-V_{ideal}\\ =[c_{A}\cdot M_{A}+c_{B}\cdot M_{B}]/\rho -[c_{A}\cdot M_{A}/\rho_{A}+c_{B}\cdot M_{B}/\rho_{B}] \end{array}$$

*V* is the real volume, and *V_ideal_* is the volume of the ideal mixture. The excess volume is further assumed to depend on the concentrations,

(46) $$\Delta V=c_{A}c_{B}V_{X}$$

with *V_X_* being a constant parameter, independent of temperature and concentration. Figure 11(b) plots the excess volume of Au-Cu alloy *versus* composition. Obviously, the excess volume reaches the minimum values at 50%, corresponding to the most pronounced volume effect, and the liquid is characterized by the highly non-ideal solution. In experiments, the influence of the excess volume on density is also verified and Equation 44 can well describe the composition dependence of density of some systems such as Cu-Ni alloys [20]. It is pointed out that the composition dependence of the density usually varies with the systems, and some liquid alloys do not exhibit strong excess volume, for instance the zero excess volume observed in Ag-Cu alloy [118]. In general, for the dilute solution, the Neumann-Kopp relation can be applied to interpolate the density to a certain extent.

### 3.4. Surface Tension

The thermophysical properties of undercooled melts are experimentally measured mainly by means of electromagnetic and aerodynamic levitation techniques. The advantages of electromagnetic levitation consist of two aspects. Firstly, the levitation state induced by the rf electromagnetic field can effectively exclude the contamination of container wall and enhance undercooling. The undercooling of many alloys can thus reach 200–300 K using the levitation technique, which is difficult under normal experimental conditions. Secondly, the eddy currents in the sample caused by the inhomogeneous electromagnetic field simultaneously heat and eventually melt the sample, even for some high melting-point alloys. In combination with noncontact diagnostic tools, this method is preferred in the study of bulk liquid alloys. For the aerodynamic levitation technique, the levitation and heating are manipulated independently, and usually the laser resource is supplied to control the heating and cooling processes by adjusting the power. The aerodynamic levitation is suitable to the measurements of semiconductors, metal oxides and some non-metal liquids especially.

The measurements of surface tension are carried out by the oscillating drop method based on the levitation techniques. The droplet surface will oscillate around the rest position with respect to the small perturbations. The restoring force driving the oscillation is provided by the surface tension of the droplet. As for the droplet levitated in terrestrial electromagnetic fields, the equilibrium shape deviates from the regular sphere. As a result, the Rayleigh frequency *ω*_R_ corresponding to the spherical droplet splits into a set of five frequencies *ω*_m_ belonging to different oscillation modes with *m* = −2, −1, 0, 1, 2, where *m* is the mode number of axisymmetric shape oscillation. In the case of the spherical droplet without the external fields, the relationship between the Rayleigh frequency *ω*_R_ and the surface tension *γ* is written as

(47) $$\omega_{R}^{2}=\frac{32\pi }{3}\frac{\gamma }{M}$$

where *M* is the mass of the droplet. Equation 47 can approximately describe the surface oscillation in the microgravity environment. For the levitated droplet in ground-based experiments, the frequency splits and droplet translation induced by the gravity fields should be taken into account.

The reported simulations of the surface tension mostly employ the method described in Figure 4. Although the operation of the approach is simple, the results show a relatively large error compared with the experimental measurements [54,119]. The calculated surface tension of liquid nickel in the temperature range of 1208–2273 K is 20–40% lower than the experimental data, and the value of liquid cobalt at the melting point is 17% larger than the experimental result [54]. Comparatively, the temperature dependence of the surface tension is in reasonable agreement with the experiments, in particular for liquid cobalt [56]. The large difference between predicted and measured values is attributed to the EAM potential used in the simulations. Chen [55] argued that the high-order terms of many body contributions were dropped in constructing the effective pair potential, which significantly affected the accuracy of predictions.

Both simulations and experiments suggest that there is a universal linear relationship for describing the temperature dependence of surface tension of pure metals,

(48) $$\gamma =\gamma_{L}+\gamma^{\prime }(T-T_{L})$$

where *γ_L_* is the surface tension at the liquidus temperature *T_L_*, *γ ′* = d*γ* d*T* is the temperature coefficient. This linear relation is also held in some binary alloys. For most liquids around us, the surface tension shows negative temperature dependence. However, we note a few exceptional cases with positive temperature dependence. From the results of Cu-Si systems reported by Egry *et al.* [120], the temperature dependence of surface tension transforms from negative to positive values when the composition exceeds 30%. Similar observations are also obtained in Zr-based binary and ternary alloys. A possible explanation is the effect of local ordering structures such as clusters of intermetallic compound on the thermodynamic properties. The simulated evidence about the abnormality is not reported and is an interesting issue for further investigation. Tables 5, 6 summarize some measured and simulated results for pure metals and binary liquid alloys.

Like the pure liquid metals, the calculated surface tension of binary alloys shows large deviation from experimental results. For example, the surface tension of Ni_50_Al_50_ alloy underestimates the available experimental values by 33–35% values at 1900–2000 K [137]; the calculated values of Ni-Cu alloys are 30% to 40% higher than the experimental data [55]. With the increase of Cu composition, the surface tension of Ni-Cu alloys monotonically decreases, which is identical to the experiments. The Ni-Cu alloy is a simple binary system and the monotonic relationship between the surface tension and the composition is in the expectation. For more complicated alloy systems that contain some intermetallic compounds in phase diagram, the composition dependence of the surface tension, or the influence of the local ordering clusters in liquids on the surface properties is an unnegligible factor.

The composition dependence of surface tension can be theoretically described by ideal solution model and Butler model approximately. In the ideal solution model, the surface tension of binary alloys is given by,

(49) $$\gamma_{AB}=C_{A}^{surf}\gamma_{A}+C_{B}^{surf}\gamma_{B}$$

where *γ_A_* and *γ_B_* are the surface tension of pure elements. *C_A_^surf^* and *C_B_^surf^* are the surface concentrations of each component, respectively, and are given by

(50a) $$C_{A}^{surf}=\frac{C_{A}}{C_{A}+C_{B}\text{exp}[-A(\gamma_{B}-\gamma_{A})/RT]}$$

(50b) $$C_{B}^{surf}=\frac{C_{B}}{C_{B}+C_{A}\text{exp}[A(\gamma_{B}-\gamma_{A})/RT]}$$

here *C_A_* and *C_B_* are the compositions of A and B elements, and *A* denotes the averaged molar surface area. In the Butler model, the surface tension of alloys is written as functions of surface and bulk partial Gibbs excess free energies, *ΔG^S^* and *ΔG^B^*

(51) $$\begin{array}{l} \gamma_{AB}=\gamma_{A}+\frac{RT}{A_{A}}\text{ln}\left(\frac{1-C_{B}^{surf}}{1-C_{B}^{Bulk}}\right)+\frac{1}{A_{A}}(\Delta G_{A}^{S}-\Delta G_{A}^{B})\\ =\gamma_{B}+\frac{RT}{A_{B}}\text{ln}\left(\frac{1-C_{A}^{surf}}{1-C_{A}^{Bulk}}\right)+\frac{1}{A_{B}}(\Delta G_{B}^{S}-\Delta G_{B}^{B}) \end{array}$$

where *A_A B_*_,_ are the surface area occupied by *A* and *B* atoms respectively, *C_A B_^Bulk^*_,_ are the bulk composition of species A and B. Comparatively, the prediction of Butler model shows closer to the experiments. For systems consisting of semiconductor elements, the modifications involving the bond energy of potential ordering clusters at surface are proved to effectively improve the predications [120]. The microstructural evolution of melts, in particular in undercooled state, significantly influences the thermodynamic appearance.

### 3.5. Diffusion

#### 3.5.1. Self-diffusion Coefficient

The self-diffusion coefficients of pure liquid alloys are readily obtained by calculating the velocity autocorrelation function or MSD as described in Equations 19 and 20. For most liquid metals and alloys, the variation of self-diffusion coefficient with temperature can be well represented by the Arrhenius relation,

(52) $$D=D_{0}\text{exp}(-\frac{Q}{RT})$$

where *D*_0_ is the pre-exponential factor and *Q* is the diffusion activation energy. The MD simulations reveal that this relation also perfectly operates in the undercooling region. Table 7 summarizes the Arrhenius fits to the simulated self-diffusion coefficients of liquid Cu, Co, Ni and Al using the EAM potential. These data are difficult to achieve in experiments. The available value of liquid copper at the melting temperature is about 0.379 × 10^−8^ m^2^s^−1^ [138], which is comparable to the simulated values.

In contrast to these liquid metals, the self-diffusion behaviors of some covalent network-forming liquids such as Si [139], SiO_2_ [140] in the undercooled region do not obey the Arrhenius law. Instead, the Vogel-Fulcher-Tamman (VFT) equation, *D* = *D*_0_ exp (− *AT*/_0_(*T −T*_0_)), and a power law, *D* = *D*_0_ (*T/T*_0_ *−* 1)*^α^*, are used to describe the temperature dependence of dynamic behaviors of these special liquids [141]. Here *A* is the parameter controlling how closely the system obeys the Arrhenius law. The liquids whose dynamic properties show the non-Arrhenius law are called “fragile” liquids.

In contrast, the “strong” liquids are referred to the Arrhenius transport. MD simulations have demonstrated that a transition from fragile to strong occurs when the glass transition is approached. The anomalies of the thermodynamic quantities are believed to accompany a liquid-liquid transition and the latter is related to the existence of an assumed spinodal line. The temperature dependence of self-diffusion coefficients of some binary alloys also exhibits non-Arrhenius behavior in undercooled region [137,142]. Pasturel *et al.* explained that the rapid icosahedral increase in the undercooled region is responsible for the non-Arrhenius dynamics slowing down [142].

#### 3.5.2. Inter-diffusion Coefficient

**Figure 12:**
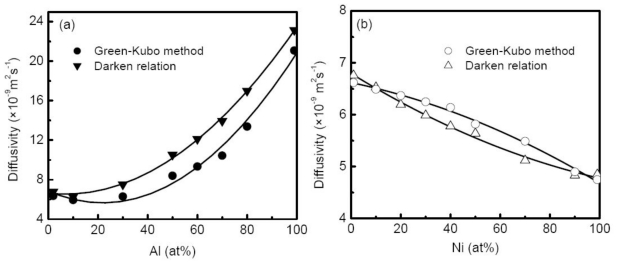
M-S diffusion coefficients as a function of concentration. (**a**) Al-Cu alloy; (**b**) Ni-Cu alloy.

From the discussion above, there are two approaches to calculate the M-S inter-diffusion coefficient in MD simulations, the Green-Kubo relation as described in Equation 27 and the Darken relation. Therefore, we can test the validation of the Darken relation in the liquid alloy by comparing the two results. Chen *et al.* [58] calculated the M-S diffusion coefficient of liquid Al-Cu alloys at 1500 K with compositions ranging from 2% to 99% Al using both Green-Kubo relation and the Darken relation based on the self-diffusion coefficient, as shown in Figure 12(a). The calculated self-diffusion coefficients of Al and Cu in Al-Cu alloys display similar concentration dependence, and the differences between them show rather small. The comparisons of the two M-S diffusion coefficients indicate that the Darken relation works well in dilute concentration region (<10% Al), whereas the M-S diffusion coefficients by the Darken relation are markedly larger than those by the Green-Kubo method for medium concentration systems, and this discrepancy reaches the maximum at 70% Al. The difference does not show significant for Ni-Cu alloys as illustrated in Figure 12(b). Obviously, the discrepancy comes from the fact that the interplay of diffusion flux is neglected in the Darken relation. For different alloy systems, the interactions between the diffusion flux of different species may discriminatively influence the M-S diffusion process, and leads the Darken relation to fail. Asta *et al.* [143] studied the transport properties of Ni-Al alloys. Similar to Chen’s results, the selfdiffusion coefficients of Ni and Al in melts are nearly identical, whereas they are higher than the interdiffusion coefficients (M-S diffusion coefficients) over the entire composition range, suggesting the failure of the Darken relation. An explanation provided by Asta *et al.* is that the Ni-Al melt is characterized by the highly non-ideal solution including the appreciable chemical short-range order (CSRO), highly negative enthalpies of mixing entropies and atomic volumes, which yields a lower inter-diffusion coefficient than that predicted by the simple weighted average argument [144]. This explanation agrees with the results of Al-Cu and Ni-Cu alloys by Chen *et al.* [58]. In both cases, the thermodynamic correction factors positively deviate from the ideal solution, and as a result the M-S diffusion coefficients from the Green-Kubo relation are lower than the prediction of the Darken relation. Contrarily, for Cu-Ni alloy, the thermodynamic correction factor negatively deviates from the ideal solution, and the corresponding M-S diffusion coefficients are universally higher than the values of the Darken relation as shown in Figure 12(b).

By means of the thermodynamic correction factor, the Fickian diffusion coefficient can be calculated through the M-S diffusion coefficient. From the results of Chen *et al.* [58], the composition dependence of the Fickian diffusion coefficient shows a non-monotonic form, which is determined by the thermodynamic correction factor, in other words, how the system deviates from the ideal solution model.

The variation of the Fickian diffusion coefficient with the temperature also follows the Arrhenius law. Table 7 lists the fitted results of Al_60_Cu_40_ and Ni_50_Cu_50_ alloys. It is noted that the Arrhenius law well describes the temperature behavior of diffusion down to the moderate undercooling for most alloys. When the temperature approaches the glass transition, the Arrhenius law shows less effective. Horbach *et al.* [144] simulated the self-diffusion coefficients of Al and Ni in Al_80_Ni_20_ melt as well as the Fickian diffusion coefficients, and the results indicated that they sharply decreased as the critical temperature of the mode-coupling theory was approached, and the temperature dependence was highly non-Arrhenius. Furthermore, the thermodynamic correction factor was found to strongly depend on temperature, in particular in the high undercooling region.

Experimental measurements of inter-diffusion coefficient in multi-component systems show more difficult because of their concentration or chemical-potential dependences of all other species, and thus the reported data is limited. In general, the self-diffusion coefficient and Fickian diffusion coefficient are measured by the long-capillary (LC) method, which can effectively suppress the natural convection and minimize the systematic error of convection contributions to the total mass transport. The studied systems focus on low melting-point alloys such as Sn- and Tin-based alloys [145,146]. Furthermore, in a realistic directional solidification environment, the inter-diffusion coefficient of Al-Cu alloy is measured *in situ* by analyzing the concentration profile ahead of a planar liquid/solid interface [147–149].

In conclusion, the self-diffusion coefficients of the two components in the binary alloys usually show close values and similar composition dependence. The Darken relation can be used to predict the inter-diffusion coefficient of dilute alloy melts, whereas it yields less credible values in the moderate concentration region due to the non-ideal solution character of the alloy melts. The Arrhenius law gives a reasonable description of the temperature dependence of various diffusion coefficients of liquid alloys in the low and moderate undercooling regions. The further understanding of the diffusion behavior of undercooled liquid alloys requires accurate knowledge of the microstructural and thermodynamic evolution of liquid alloys.

### 3.6. Viscosity

The validity of the NEMD method for calculating the viscosity always attracts the researchers interests during its development. Holian and Evans [150] examined the shear viscosities of LJ liquids produced by NEMD and Green-Kubo methods and revealed that the disagreement between them near the triple point originates from the abnormally large tail of the shear-stress autocorrelation function, and they are in good agreement far from this region. For the liquid aluminum, the comparisons between NEMD and EMD show an excellent agreement from 935 to 1175 K [151]. However, the systematic difference in liquid nickel using the two methods is up to 20%, and the difference intends to reduce in low viscosity systems. Cherne and Deymier [152] ascribed the difference to the thermostate used in the NEMD, whereas there is no further evidence to support this conclusion. Contrarily, in the calculations of shear viscosity of liquid copper, EMD, NEMD and RNEMD produce good consistence over the temperature range from 900 to 1700 K [59], and moreover well agree with the experimental measurements, as listed in Table 8. Essentially, the accuracy of calculated viscosities depends on the potentials. Koishi *et al.* [153] employed the pseudopotential method, tight-binding method and empirical potential to calculate the viscosities of Na and Fe, and the results varied from quantitative to qualitative levels.

The temperature dependence of viscosity shows more complicated compared to density and surface tension. For most alloys in moderate undercooling region, the temperature dependent viscosity can be described by Arrhenius law

(53) $$\eta =\eta_{0}\text{exp}\left(\frac{\Delta E}{\text{R}T}\right)$$

where *η*_0_ is the pre-exponential factor, *ΔE* is the activity energy and R is the gas constant. Reference 154 lists the values of *η*_0_ and *ΔE* for some typical pure metals. Alternatively, Cohen and Turnbull proposed the VFT relation based on the free volume model [155],

(54) $$\eta =\eta_{0}\text{exp}\left(\frac{{AT}_{0}}{T-T_{0}}\right)$$

where *T*_0_ is the glass transition temperature, and *A* is a constant. The third relation for describing the temperature dependence of viscosity is the power law, which is developed from the mode coupling theory (MCT) [156],

(55) $$\eta =\eta_{0}{\left(\frac{T}{T_{0}}-1\right)}^{-\alpha }$$

In fact, the three relations describe different dynamic behaviors of undercooled liquids. If the macroscopic dynamics properties such as diffusion and shear viscosity or structural relaxation time obey the Arrhenius law, the liquid is generally called “strong”. It reflects a fast structural relaxation process and features the most undercooled alloys. The Arrhenius law cannot accurately describe the dynamic behavior when the undercooled liquid approaches the glass transition temperature, and the temperature dependence in the region intends to follow the VFT relation. In contrast to the “strong” liquid, the liquid ruled by the VFT relation is called “fragile”. For some special melts such as Si [139] and SiO_2_ [140], a dynamic transition from the “fragile” to the “strong” is predicted theoretically as a result of the liquid-liquid phase transition in those undercooled covalent liquids, which is first proposed for explaining the anomalous thermodynamic properties of undercooled water near 228 K.

Lazarev *et al.* [157] simulated the self-diffusion coefficients and viscosities of Ag-Cu alloy from high temperature above the melting point to the region near the glass transition. The temperature dependence of viscosity shows a high non-Arrhenius behavior in the high undercooling region, suggesting a typical fragile liquid as approaching the glass transition. Due to the significant slow dynamic relaxation of high undercooled liquids, the calculation of dynamic properties shows rather difficult and the error is also inevitably enlarged. The NEMD provides a reliable manner to study the dynamic behavior near the glass transition in detail. For Ag-Cu alloy, the predicted divergence temperature for viscosity is comparable to the Kauzmann temperature from the thermodynamic properties, which implies a possible thermodynamic origin of the dynamic abnormalities. They also argued that the Stokes-Einstein law breaks down near the glass transition. Han *et al.* discussed the problem in liquid copper [59]. The Stokes-Einstein law describes the correlation between the viscosity and self-diffusion as,

(56) $$D=\frac{k_{b}T}{6\pi \text{R}\eta }$$

Another similar equation, Sutherland-Einstein law differs in the pre-factor,

(57) $$D=\frac{k_{b}T}{4\pi \text{R}\eta }$$

Therefore, the universal relation is written as

(58) $$\frac{D\text{R}\eta }{k_{b}T}=\text{const}$$

The constant is equal to 1/6π for Stokes-Einstein law and 1/4π for Sutherland-Einstein law. According to Han’s simulations [59], this constant is about 0.099, which is larger than Stokes-Einstein and Sutherland-Einstein laws’ results and close to 1/3π. This conclusion needs further simulations in more metals and alloys.

Moelwyn-Hughes developed a simple model to set up the composition dependence of viscosity for a binary system [158],

(59) $$\eta =(x_{A}\eta_{A}+x_{B}\eta_{B})(1-2x_{A}x_{B}\Omega /\text{R}T)$$

where *η_A_* and *η_B_* are viscosities of elements, *x_A_* and *x_B_* the mole fractions, *Ω* is the regular solution interaction parameter. The model seems to successfully predict the viscosity of some metal systems, whereas fails to give a prediction of low viscosity behavior at the eutectic compositions. The corrections for the atomic size are proven to be necessary.

## 4. Conclusions

This paper reviews recent achievements of thermophysical properties of liquid metals and alloys by molecular simulations. The simulation methods of density, specific heat, surface tension, diffusion and viscosity of undercooled metals are summarized. The simulated results are compared with experimental data, and the possible factors that influence the results are discussed.

The accuracy of the simulated results relates closely to the potential function, and also the computation model. For the potential model, the EAM and MEAM are widely applied in metal and alloy systems. They have proven to be capable of reasonably describing the thermodynamic and structural characteristics of liquid metals and alloys. The adaptability of EAM and MEAM depends on the appropriate parameters that are determined by fitting experimental or first-principle results, and most of them refer to crystal structure. Therefore, it is necessary to improve the potentials to be more suitable for the liquid and metastable liquid systems. With the development of computation capability, large-scale MD simulations are more frequently employed. The calculations of thermophysical properties and phase transition with *ab initio* simulations are also becoming possible.

A number of simulated results show that the temperature dependences of enthalpy, density and surface tension, follow linear relations for most liquid metal systems, even in the undercooled region. The specific heat of the melts is more or less dependent on temperature. Compared with experimental data, the melting temperature, specific heat and density calculated by MD, generally show good agreements. The variations of thermophysical quantities with composition are more complicated, depending on the characteristics of the alloy. The ideal solution model can only account for the thermophysical behavior of a few alloy systems. Most alloys exhibit the mixing effect, and the excess volume, enthalpy and entropy of mixing are unnegligible. The temperature and composition dependences of the thermophysical properties provide us indications in analyzing the structural features of undercooled liquids corresponding to various thermodynamic experiences and find out the essential correlation between them. For most pure metals and alloys, the variations of both the diffusion coefficients and the inter-diffusion coefficients with temperature follow the Arrhenius law down to a moderate undercooling region. Similar to many thermodynamic properties, the validity of the Darken relation for inter-diffusion coefficients relies on the nature of the alloy, which is proven invalid for strong non-ideal solutions. The EMD, NEMD and RNEMD methods for calculating dynamic viscosity are consistent and reliable. The simulated results near the glass transition demonstrate a non-Arrhenius behavior of viscosity, revealing a crossover from strong to fragile when liquids are undercooled upon the glass transition. MD simulations provide us a promising tool in studying the dynamic abnormalities in the high undercooling region, as well as their relations to the thermodynamic behaviors.

## Figures and Tables

**Table 1:** Melting points obtained from MD simulations and comparison with experiments.

	$T_{m}^{Calc.}$ (K)	$T_{m}^{Exp.}$ (K)	Deviation
Cu [41]	1,320.5 ± 1.5	1,356	−2.62%
Au [41]	1,182.5 ± 1.5	1,336	−11.49%
AuCu_3_ [41]	1,240.5 ± 1.5	1,250	−0.76%
AuCu [41]	1,173.5 ± 0.5	1,185	−0.97%
Au_3_Cu [41]	1,151.5 ± 1.5	1,220	−5.61%
Ni_3_Al [46]	1,705	1,663	2.5%
	1,725		3.8%
Al_50_Ni_50_ [106]	1,520	1,920	−20%

**Table 2:** Enthalpy of liquid metals as a function of temperature.

Enthalpy *H* (J mol^−1^)	
Co	*H* = −4.09 ×10^5^ + (32.51 ± 0.19) ·*T* [40]
	*H* = −4.21×10^5^ + (38.60 ± 0.08) ·*T* [40]
Cu	*H* = −3.45 ×10^5^ + (33.68 ± 0.19) ·*T* [41]
Au	*H* = −3.81×10^5^ + (34.30 ± 0.38) ·*T* [41]
Ti	*H* = − 4.661×10^5^ + 39.193 · *T* − 2.430 × 10^−3^ · *T*^2^ [39]
Al	*H* = −3.197 × 10^5^ + 35.303 × 10^−4^*T* − 2.68 × 10^−3^ · *T*^2^ · [39]

**Table 3:** Specific heat of liquid metals.

	Simulated Values (J mol^−1^ K^−1^)	Experimental Values (J mol^−1^ K^−1^)
Co	32.509 ± 0.194 [40]	40.38 *T* > 1768 K [110]
	38.595 ± 0.084 [40]	40.6 1541 K < *T* < 1768 K [21]
Cu	33.68 ± 0.19 [41]	31.5 1356 K < *T* < 1873 K [111]
Au	34.30 ± 0.38 [41]	29.3 1336 K < *T* < 1673 K [111]
Ti	*C_P_* = 39.1991–4.8650 × 10^−3^ · *T* [39]	33.53 *T* = 1933 K [111]
Al	*C_P_* = 35.3091–5.3544 × 10^−3^ · *T* [39]	31.8 *T* = 933 K [111]
Ag	*C_P_* = 30.5357–4.8555 × 10^−3^ · *T* [112]	30.5 1233.7 K < *T* < 1573 K [111]

**Table 4:** Density of typical liquid metals and alloys.

	Simulated values		Experimental values	
	
	*ρ_m_* (×10^3^ kg/m^3^)	*∂ρ/∂T* (×10^−1^ kg m^−3^K^−1^)	*ρ_m_* (×10^3^ kg/m^3^)	*∂ρ/∂T* (×10^−1^ kg m^−3^K^−1^)
Co	7.74	−7.70 [40]	7.76	−10.9 [111]
	7.49	−9.17 [40]		
Cu	7.868	−8.91 [41]	8.00	−8.0 [111]
Au	16.85	−24.15 [41]	17.36	−15 [111]
Ti	4.55	−2.16 [39]	4.13	−2.3 [111]
Al	2.55	−1.18 [39]	2.385	−3.5 [111]
Cu_75_Au_25_	10.98	−12.39 [41]	11.39	−19.5 [115]
Ag_80_Cu_20_	-	-	9.0	−6 [116]
Ni_11_Cu_89_	-	-	7.97	−7.95 [20]
Si_10_Cu_90_	-	-	7.591	−6.308 [117]

**Table 5:** Surface tension of typical pure metals.

Element	*T*_m_(K)	*γ*_0_ (N·m^−1^)	d*γ*/d*T* (×10^−4^ N·m^−1^·K^−1^)	Ref.
Cu	1357	1.30	−2.64	Egry *et al.* [117]
		1.29	−2.34	Egry *et al.* [19]
Ni	1726	1.764	−3.30	Wei *et al.* [57]
		1.770	−3.30	Egry *et al.* [19]
Al	933	0.88	−2.0	Egry *et al.* [121]
		1.024 ± 0.048	−2.74 ± 0.25	Sarou-Kanian *et al.* [122]
Fe	1811	1.92	−3.97	Egry *et al.* [19]
		1.888±0.031	−2.85 ± 0.15	Wille *et al*. [30]
Ag	1235	0.91	−1.8	Egry *et al.* [123]
Au	1337	1.149	−1.4	Egry *et al.* [13]
		1.875	−3.48	Wei *et al.* [23]
Co	1773	1.881	−3.4	Keene [124]
		1.887	−3.3	Egry *et al.* [125]
Si	1683	0.784	−6.5	Przyborowski *et al.* [126]
		0.73	−6.2	Fujii *et al.* [127]

**Table 6:** Surface tension of selected binary undercooled alloys.

Alloys (at%)	*T*_L_ (K)	*γ*_0_ (N·m^−1^)	d*γ*/d*T* (×10^−4^ N·m^−1^·K^−1^)	Ref.
Ni_90.1_Si_9.9_	1623	1.697	−3.97	Wei *et al.* [25,57,128]
Ni_70.2_Si_29.8_	1488	1.693	−4.23	
Ti_49_Al_51_	1753	1.094	−1.422	Wei *et al.* [129]
Co_93_Mo_7_	1744	1.895	−3.1	Wei *et al.* [130]
Co_75_Si_25_	1607	1.604	−4	Wei *et al.* [131]
Co_80_Pd_20_	1613	1.687	−1.5	Egry *et al.* [132]
Zr_64_Ni_36_	1283	1.54	1.07	Egry *et al.* [18]
Cu_70_Co_30_	1638	1.22	−2.9	Egry *et al.* [125]
Co_50_Fe_50_	1752	1.8	−3.72	Egry *et al.* [133]
Cu_90_Si_10_	1246	1.332	−2.686	Egry *et al.* [120]
Fe_80_Ni_20_	1748	2.056	−0.15	Egry *et al.* [134]
Au_56_Cu_44_	1183	1.21	−0.15	Egry *et al.* [135]
Cu_90_Ni_10_	1409	1.31	−2.21	Egry *et al.* [136]

**Table 7:** Arrhenius fits to self-diffusion and inter-diffusion coefficients of liquid metals and alloys.

	Simulations	
	*D*_0_ (m^2^s^−1^)	*Q* (kJ·mol^−1^)
Cu	6.69 × 10^−8^	34.2 [134]
	7.38 × 10^−8^	42.0 [59]
Ni	9.69 × 10^−8^	47.1 [134]
Co	1.29 × 10^−7^	48.8 [40]
Al	1.78 × 10^−7^	25.8 [134]
Ni(Ni_50_Al_50_)	1.01 × 10^−7^	45.3 [137]
Al(Ni_50_Al_50_)	0.94 × 10^−7^	46.3 [137]
Al_60_Cu_40_	9.16 × 10^−8^	17.0 [58]
Ni_50_Cu_50_	8.03 × 10^−8^	48.9 [58]

**Table 8:** Arrhenius fits to viscosity of liquid copper.

Methods	*η*_0_ (mPa·s)	*E_a_*/*k_b_* (K)
NEMD	4.09301	2591.23024
RNEMD	3.94354	2624.62262
EMD	3.88996	2570.48062
Experimental	4.35932	2867.86973
